# Toxicity of Silver–Chitosan Nanocomposites to Aquatic Microcrustaceans *Daphnia magna* and *Thamnocephalus platyurus* and Naturally Luminescent Bacteria *Vibrio fischeri*

**DOI:** 10.3390/nano14141193

**Published:** 2024-07-12

**Authors:** Mariliis Sihtmäe, Jüri Laanoja, Irina Blinova, Anne Kahru, Kaja Kasemets

**Affiliations:** 1 Laboratory of Environmental Toxicology, National Institute of Chemical Physics and Biophysics, Akadeemia tee 23, 12618 Tallinn, Estonia; juri.laanoja@taltech.ee (J.L.); irina.blinova@kbfi.ee (I.B.); anne.kahru@kbfi.ee (A.K.); kaja.kasemets@kbfi.ee (K.K.); 2 Department of Chemistry and Biotechnology, School of Science, Tallinn University of Technology, Ehitajate tee 5, 19086 Tallinn, Estonia; 3 Estonian Academy of Sciences, Kohtu 6, 10130 Tallinn, Estonia

**Keywords:** chitosan, silver, antimicrobial nanomaterials, environmental hazard evaluation

## Abstract

All novel materials should be analyzed for their potential environmental hazard. In this study, the toxicity of different silver–chitosan nanocomposites—potential candidates for wound dressings or antimicrobial surface coatings—was evaluated using environmentally relevant aquatic microcrustaceans *Daphnia magna* and *Thamnocephalus platyurus* and naturally luminescent bacteria *Vibrio fischeri*. Three silver-chitosan nanocomposites (nAgCSs) with different weight ratios of Ag to CS were studied. Citrate-coated silver nanoparticles (nAg-Cit), AgNO_3_ (ionic control) and low molecular weight chitosan (LMW CS) were evaluated in parallel. The primary size of nAgCSs was ~50 nm. The average hydrodynamic sizes in deionized water were ≤100 nm, and the zeta potential values were positive (16–26 mV). The nAgCSs proved very toxic to aquatic crustaceans: the 48-h EC_50_ value for *D. magna* was 0.065–0.232 mg/L, and the 24-h LC_50_ value for *T. platyurus* was 0.25–1.04 mg/L. The toxic effect correlated with the shedding of Ag ions (about 1%) from nAgCSs. Upon exposure of *V. fischeri* to nAgCSs for 30 min, bacterial luminescence was inhibited by 50% at 13–33 mg/L. However, the inhibitory effect (minimum bactericidal concentration, MBC) on bacterial growth upon 1 h exposure was observed at higher concentrations of nAgCSs, 40–65 mg/L. LMW CS inhibited bacterial luminescence upon 30-min exposure at 5.6 mg/L, but bacterial growth was inhibited at a much higher concentration (1 h MBC > 100 mg/L). The multi-trophic test battery, where *D. magna* was the most sensitive test organism, ranked the silver-chitosan nanocomposites from ‘extremely toxic’ [L(E)C_50_ ≤ 0.1 mg/L] to ‘very toxic’ [L(E)C_50_ > 0.1–1 mg/L]. Chitosan was toxic (EC(L)_50_) to crustaceans at ~12 mg/L, and ranked accordingly as ‘harmful’ [L(E)C_50_ > 10–100 mg/L]. Thus, silver-chitosan nanocomposites may pose a hazard to aquatic organisms and must be handled accordingly.

## 1. Introduction

The widely used biocidal/antimicrobial nanomaterials such as nanosilver, CuO and ZnO are toxic not only to target organisms (e.g., bacteria, fungi) but also to non-target species such as crustaceans, algae and fish. Moreover, according to their sensitivity patterns, aquatic species are often more threatened than the target species (pathogenic microbes). Notably, nanosilver is the most toxic, widely produced, and used biocidal nanomaterial [1]. Despite that, the ecotoxicological aspects of silver-containing antimicrobial or antifouling products have been insufficiently studied [2]. According to the European Chemicals Agency database, silver registered under the REACH Regulation [3] is manufactured in and/or imported to the European Economic Area at remarkable amounts, ≥10,000 to <100,000 tons per year [4]. According to the bibliometric survey conducted by Juganson et al. 2015 [1], the biggest share (31%) of the total use of AgNPs can be attributed to the production of sensors (31%), followed by the use as antimicrobials (17%) and for catalysis (12%). Moreover, silver compounds are increasingly used in healthcare, such as wound dressings, bandages, catheter coatings, and disinfectants [5]. Indeed, due to the broad spectrum and variety of mechanisms of antimicrobial action of silver, there is a low risk of developing antimicrobial resistance (AMR), different from common antibiotics [6,7]. In addition, nanosilver is also used in consumer products such as sports textiles, detergents, and deodorants [8]. This warrants the need to evaluate the adverse effects of silver-containing materials on environmental species.

A specific type of silver-based antimicrobials is silver-chitosan nanocomposites developed, e.g., via incorporating silver nanoparticles into chitosan-based hydrogels for wound dressings as well as topical skin formulations that have shown enhanced effects in eradicating resistant bacteria and accelerating wound healing [9]. Chitosan is a promising, non-toxic, biocompatible material used not only for biomedical applications but also in various other areas. More specifically, chitosan is approved by the U.S. Food and Drug Administration [10], and there are several commercially available chitosan-based products, e.g., for wound treatment (ChitoSAM 100, ChitoHeal, ChitoRhino, Chitoderm plus, etc.). The main application areas of chitosan were recently mapped by Thambiliyagodage et al. (2023) [11] and Wang and Zhuang (2022) [12] as follows: agriculture, wastewater treatment, food and textile industry, cosmetics, and medicine.

According to our bibliometric search on the number of scientific papers in the Web of Science database (as of 21 March 2024) concerning the research of silver-chitosan (nano)composites in the above-listed six areas by adding one more search term ‘antimicrobial*’, the biggest share of papers was found in the field of antimicrobials (1806 papers in total), followed by medicine (525 papers), wastewater treatment (156), agriculture (132), food industry (121), textile industry (44), and cosmetics (39).

However, for the successful commercialization of any new material, besides efficacy and human toxicology, data on its environmental safety are obligatory. Indeed, as all produced materials will inevitably reach the environment through various waste streams during their lifecycle, some applications directly impact the environment (such as agriculture and wastewater treatment), so assessing the ecotoxicological effects of silver-chitosan nanocomposites is crucial.

The main aim of the current study was to evaluate the potential environmental hazard of three different types of silver–chitosan nanocomposites (nAgCSs) synthesized in our laboratory as promising antimicrobial nanomaterials for wound dressings and surface coatings. For comparison, citrate-coated silver nanoparticles (chitosan-free control), AgNO_3_ (ionic control) and low molecular weight chitosan were tested in parallel. Acute toxicity assays with three aquatic test species belonging to different trophic levels were used: naturally luminescent bacteria *Vibrio fischeri* (destructors) and planktonic microcrustaceans *Thamnocephalus platyurus* and *Daphnia magna* (consumers). To our knowledge, this is the first study evaluating silver-chitosan nanocomposites’ toxicity to the aquatic species.

## 2. Materials and Methods

### 2.1. Synthesis and Characterization of Ag-Chitosan Nanocomposites

Silver-chitosan nanocomposites (nAgCSs) were synthesized by the reduction of silver nitrate (AgNO_3_) with trisodium citrate (Na_3_C_6_H_5_O_7_; TSC) and stabilized by low molecular weight chitosan (50–190 kDa). Altogether, three nAgCSs with different weight ratios of silver-chitosan were synthesized: (i) 1:0.3 (nAgCS-0.3), (ii) 1:1 (nAgCS-1) and (iii) 1:3 (nAgCS-3). All the chemicals used to synthesize nAgCSs and citrate-coated silver NPs (nAg-Cit) were purchased from Sigma-Aldrich (Hamburg, Germany) and were of chemical purity ≥98%.

Briefly, for nAgCSs synthesis, AgNO_3_ (1 g Ag/L), TSC (10 g/L) and citric acid (10 g/L) solutions were prepared in deionized (DI) water (Millipore Milli-Q^®^, Merck Life Science BV, Hoeilaart, Belgium) and filtrated through a 0.2 µm pore-sized syringe filter (Sarstedt, Nümbrecht, Germany). Then, 10 mL of AgNO_3_ was added to 56 mL DI water and heated to boiling on the magnetic stirrer (Heidolph, Schwabach, Germany). Subsequently, 4 mL of TSC was added, and the mixture was kept at ~100 °C, 250 rpm, for the next 10 min to facilitate the formation of AgNPs (citrate-stabilized AgNPs). The suspension was cooled to room temperature, pH regulated to ~4.8 by 1% citric acid, and chitosan was added under stirring at 200 rpm. Chitosan was dissolved beforehand in 1% (vol/vol) acetic acid at the concentration of 10 g/L and allowed for complete dissolution at room temperature for at least 1–2 days before nAgCS synthesis. Before use, chitosan was diluted ten times in DI water (1 g/L) and filtered through a 0.45 µm filter (Sarstedt, Nümbrecht, Germany). All synthesized nAgCSs were ultrasonicated by a probe sonicator (Branson Digital Sonifier^®^, Emerson, Marietta, OH, USA) for 10 s at 10% of maximum power (400 W) and dialysed against 2 L of DI water for 48 h (changing the DI water after 24 h) using dialysis tubes with cut-off 12 kDa (Viskase, Lombard, IL, USA). The concentration of Ag in nAgCS was measured by atomic absorption spectroscopy (AAS) (Analytic Jena, contrAA800, Jena, Germany). Synthesized nAgCSs were stored at ~4 °C in the dark for up to six weeks. The nAgCS were analyzed for shape and size using transmission electron microscopy in scanning mode (STEM) in a Titan Themis 200 (FEI) microscope (Thermo Fisher Scientific, Waltham, MA, USA), a service commissioned by Tartu University, Estonia. The primary sizes of nAgCs were determined by measuring their diameter in the respective STEM images using ImageJ 1.51j8 software.

### 2.2. Hydrodynamic Size, ξ-Potential and Stability Measurements

Dynamic Light Scattering (DLS) and Electrophoretic Light Scattering (ELS) were used to characterize the silver-chitosan nanocomposites. For the analysis, nAgCSs were suspended in DI water or toxicity testing media (2% NaCl for bacteria *Vibrio fischeri* and artificial fresh water, AFW, for microcrustaceans). Zetasizer Nano ZS and Zetasizer software 8.01 (Zetasizer Nano-ZS, Malvern Instruments Ltd., Malvern, UK) were used for the analysis. Nanocomposites’ average hydrodynamic size (d*_H_*), surface charge (ζ-potential), the suspension’s polydispersity index (PDI) and stability were measured at nAgCSs concentration of 10 mg Ag/L in disposable cuvettes (DTS1061 folded capillary zeta cell or DTS0012 square polystyrene cuvette, Malvern Instruments Ltd., Malvern, UK) at 25 °C. The stability of nanocomposite suspensions was assessed quantitatively using light scattering/mean count rate measurements and qualitatively by observation of visual settling in cuvettes.

### 2.3. Solubility/Dissolution

The concentration of silver ions shed from the nAgCSs in DI water and toxicity testing media (2% NaCl for bacteria and artificial freshwater, AFW, for microcrustaceans) was measured at 1 mg Ag/L. A 12-well plate (Falcon) containing 3 mL of each diluted sample per well was incubated at ambient temperature (~20 °C) for 1 h, 24 h, and 48 h to mimic the conditions of the conducted toxicity tests (medium composition, time, temperature). After incubation, 2.7 mL of each sample was transferred into the Vivaspin 6 (10 000 MWCO PES) filtration tube (Sartorius Stedim Biotech GmbH, Goettingen, Germany) and centrifuged for 40 min at 6225× *g*, 20 °C (Sigma 3-16PK, Sigma-Aldrich, Hamburg, Germany) to remove the non-soluble fraction of the nanocomposites. After centrifugation, 65% nitric acid (Honeywell, Charlotte, NC, USA), equivalent to 5% of the sample (filtrate) volume, was added, and the concentration of Ag in eluate/filtrate was measured by AAS (Analytic Jena, contrAA 800, Jena, Germany).

### 2.4. Toxicity Tests with Aquatic Microcrustaceans

Acute toxicity tests with aquatic microcrustaceans *Thamnocephalus platyurus* and *Daphnia magna* were performed according to ISO and OECD test guidelines [13,14] in artificial freshwater (AFW) containing (per L) 294 mg CaCl_2_⋅2H_2_O, 123.25 mg MgSO_4_⋅7H_2_O, 64.75 mg NaHCO_3_ and 5.75 mg KCl, pH = 7.8 ± 0.2. Briefly, the organisms used for the testing were hatched from dormant eggs. Both the ephippia of *D. magna* and cysts of *T. platyurus* were purchased from MicroBioTests, Inc. (Gent, Belgium) and hatched according to producer protocols [15]. In the acute immobilization tests with *D. magna* (water flea), the neonates less than 24 h old obtained by the hatching of ephippia were exposed to tested compounds at 21 °C in the dark for 48 h. In the acute mortality test with the crustacean *T. platyurus* (fairy shrimp), the larvae hatched from the cysts were exposed at 25 °C in the dark for 24 h. The tests were performed thrice in four (*D. magna*) and three (*T. platyurus*) replicates.

### 2.5. Bioluminescent Inhibition Tests with Bacteria Vibrio fischeri

Acute bioluminescence inhibition assay (exposure time 30 min) with naturally bioluminescent bacteria *Vibrio fischeri* was performed at ambient temperature (20 °C) on white sterile 96-well polypropylene microplates (Greiner Bio-One GmbH, Frickenhausen, Germany) following the Flash assay protocol [16]. The bacterial suspension used for the toxicity measurements was prepared from freeze-dried bacteria (Aboatox, Turku, Finland). All tested nanocomposite suspensions and chemicals were prepared and tested in 2% NaCl (optimal environment for the marine bacteria *V. fischeri*). Briefly, 100 μL of the test solution was pipetted into each well, which was supplemented with 100 μL of bacterial suspension by automatic dispensing in a Microplate Luminometer Orion II (Berthold Detection Systems, Pforzheim, Germany) controlled by the Simplicity 4.2 Software. All the chemicals were tested at a minimum on three different days, in 5–7 dilutions, in two replicates for each chemical. The controls, both negative (2% NaCl) and positive (ZnSO_4_, prepared from ZnSO_4_x7H_2_O, Alfa Aesar, Hamburg, Germany), were included in each run.

After the bioluminescence inhibition assay, a spot test was performed [17]. Briefly, bacteria were further incubated at 20 °C and 3 µL of bacterial suspension was pipetted as a spot on Beneckea Harvey (BH) nutrient agar medium (per L: yeast extract 3 g, tryptone 5 g, glycerol (99%) 2 mL, NaCl 30 g Na_2_HPO_4_·12H_2_O 9.45 g, KH_2_PO_4_ 1 g, (NH_4_)_2_HPO_4_ 0.5 g, MgSO_4_·7H_2_O 0.3 g, agar 15 g) after 1 and 24 h of exposure. The inoculated agar plates were then incubated in the dark for 48 h at room temperature, and the minimum bactericidal concentration (MBC) of the tested compounds was visually determined.

### 2.6. Statistical Methods

The toxicity values (E(L)C_50_) were determined from the dose–response curves using the log-normal model with Microsoft Excel 2019 macro REGTOX [18] and the statistical differences. To determine statistically significant differences between the EC_50_ values, a *t*-test in Microsoft Excel 2019 was used. Statistical significance was accepted at *p* ≤ 0.05.

## 3. Results and Discussion

### 3.1. Synthesis and Characterization of Studied Nanomaterials

Three different silver-chitosan nanocomposites (nAgCSs) were synthesized using low molecular weight chitosan (50–190 kDa) by the reduction of silver nitrate by trisodium citrate with the weight ratios of silver-chitosan 1:0.3 (nAgCS-0.3), 1:1 (nAgCS-1) and 1:3 (nAgCS-3). For comparison, citrate-coated silver nanoparticles (chitosan-free control) and AgNO_3_ (ionic control) were characterized and analyzed for their toxicity in parallel (Table 1 and Table 2).

**Figure 1:**
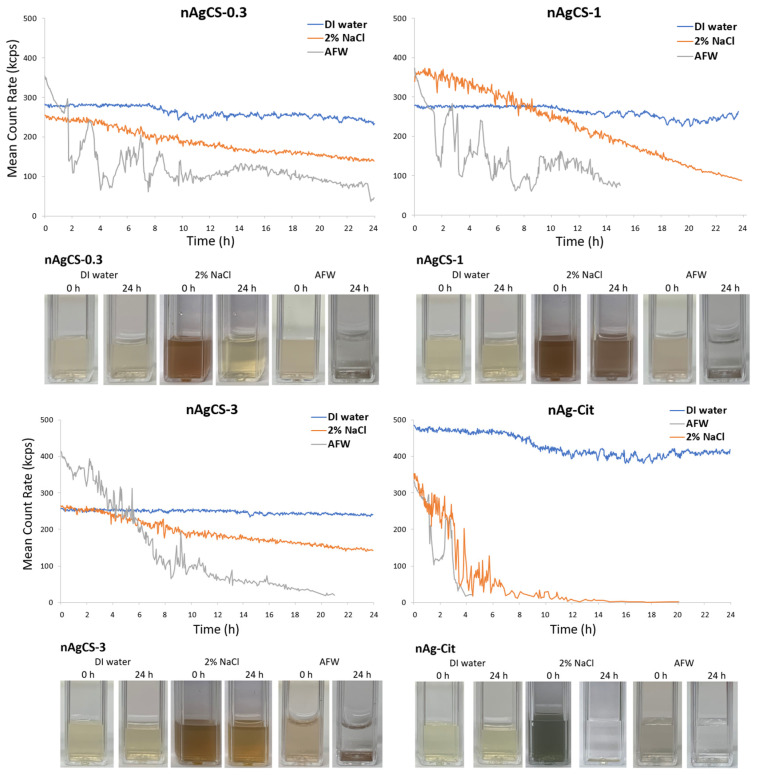
Quantitative (using light scattering/mean count rate; Zetasizer Nano-ZS, Malvern Instruments Ltd., Malvern, UK) and qualitative (visualization of the settling in cuvettes) determination of the stability of studied nanomaterials suspensions during 24 h in DI-water and toxicity testing media (2% NaCl and artificial fresh water, AFW) at 10 mg Ag/L.

The primary size of nAgCSs and nAg-Cit determined using STEM was ~50 nm [19]. The hydrodynamic size of the studied nanomaterials in DI-water varied from 37 nm (nAg-Cit) to 79–102 nm (nAgCSs). In 2% NaCl, the hydrodynamic size was generally bigger than in DI water, increasing up to 248 nm. In artificial freshwater, the hydrodynamic diameter of studied nAgCSs increased, and agglomerates exceeded 1000 nm (Table 1, Figure 1). This was probably due to the presence of divalent cations that have been shown to increase the agglomeration of AgNPs [20]. Although the agglomeration and stability of studied nanomaterials in test media (2%NaCl and AFW) were remarkably different, the shedding of silver-ions from these nanomaterials was similar, ranging from 1.6–3.6% in NaCl and 0.5–1.7% in AFW (Table 1).

The zeta potential is another critical parameter that gives information about the stability and interaction of nanomaterials with biological systems. In our study, the zeta potential of nAgCSs was positive (16–26 mV). In contrast, nAg-Cit had a negative surface charge (−36 mV) (Table 1). In DI water, the ζ-potential of chitosan (1000 mg/L, pH ~4.0) was +62 mV. Therefore, the suspension of nAgCSs in DI water could be considered stable (ζ-potential ~30 mV) [21,22]. The positive zeta potential of nAgCSs may enhance their interaction with negatively charged bacterial cell membranes, leading to increased toxicity [23]. Conversely, the negative charge of nAg-Cit may result in reduced interactions and, consequently, lower toxicity.

The stability of nAgCSs and nAg-Cit was assessed quantitatively using light scattering/mean count rate measurements and qualitatively by observation of visual settling in cuvettes (Figure 1). Both nAgCSs and nAg-Cit remained stable in DI water (Figure 1), showing no visible signs of precipitation even after 6 months of storage at 4 °C in the dark. Interestingly, in 2% NaCl, nAgCSs were more stable after 24 h settling time than nAg-Cit, which settled within 2–4 h. In AFW, all studied nanomaterials formed agglomerates with hydrodynamic sizes >1 μm (Table 1) and settled within the first 2–4 h of the experiments (Figure 1).

### 3.2. Toxicity of Studied Compounds to Bacteria Vibrio fischeri

*V. fischeri*, also known as *Aliivibrio fischeri* [24] and/or *Photobacterium phosphoreum* [25], are naturally bioluminescent, Gram-negative marine bacteria. The bioluminescence of these bacteria is closely related to their energetic metabolism, which, in turn, depends on the integrity of the bacterial cellular membrane, which is crucial for maintaining healthy cells. Thus, decreased bioluminescence reflects the inhibition of bacterial metabolic activity and is proportional to the toxicity of the studied compounds [26]. Importantly, the *V. fischeri* bioluminescence inhibition test results correlate well with the results of the other aquatic toxicity assays for a wide range of toxicants [27], and these data are often used for building QSAR models to predict the toxicity of chemicals based on their chemical structure [28].

**Figure 2:**
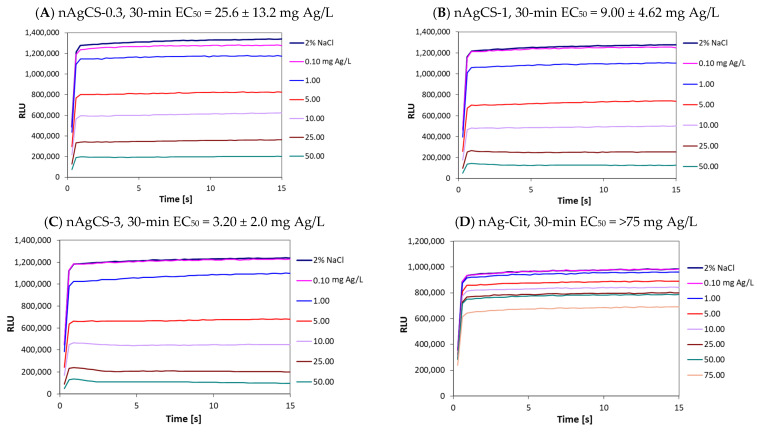

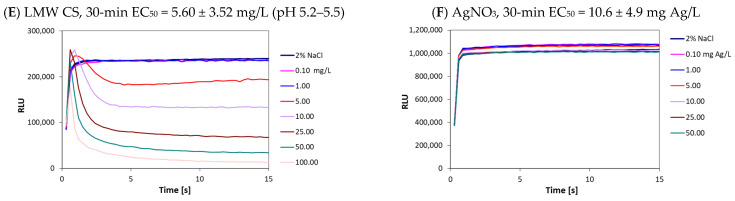
Kinetics of bioluminescence during the first 15 s of exposure of *Vibrio fischeri* to suspensions of silver-chitosan nanocomposites with silver-chitosan weight ratios of (**A**) 1:0.3 (nAgCS-0.3), (**B**) 1:1 (nAgCS-1) and (**C**) 1:3 (nAgCS-3), (**D**) citrate-coated silver nanoparticles (nAg-Cit), (**E**) low molecular-weight chitosan (LMW CS) and (**F**) silver ions (AgNO_3_). NaCl (2%) served as a control and diluent. Concentrations of test compounds are nominal. Chitosan was tested at an initial pH ranging from 4.3 (100 mg/L) to 6.5 (0.1 mg/L), and at the level of obtained 30-min EC_50_ value, the pH of chitosan was 5.2–5.5. RLU—relative light units. 30-min EC_50_ values taken from Table 2 are added to the panels. Please note that the EC_50_ values for chitosan are presented as mg compound/L and Ag-containing compounds as mg Ag/L.

Although the recommended contact time for the test sample and *V. fischeri* bacteria is 30 min, inhibition of bacterial luminescence can already be observed within the first seconds of exposure to a toxicant, depending on the type of the chemical: metals typically take longer times to show effects, whereas organic chemicals, which usually act through narcotic mechanism [28], react more rapidly [29]. The kinetics of bacterial bioluminescence during the first 15 s of the contact of bacteria with different concentrations of studied nAgCSs, nAg-Cit, AgNO_3_ and low molecular weight chitosan (LMW CS) are depicted in Figure 2. Regarding nAgCSs and nAg-Cit, the effect of increased concentration on the value of the bioluminescence signal (decrease in values) was apparent, but this was mainly due to the dark colour of the samples interfering with the luminescence endpoint (Figure 2A–D).

**Figure 3:**
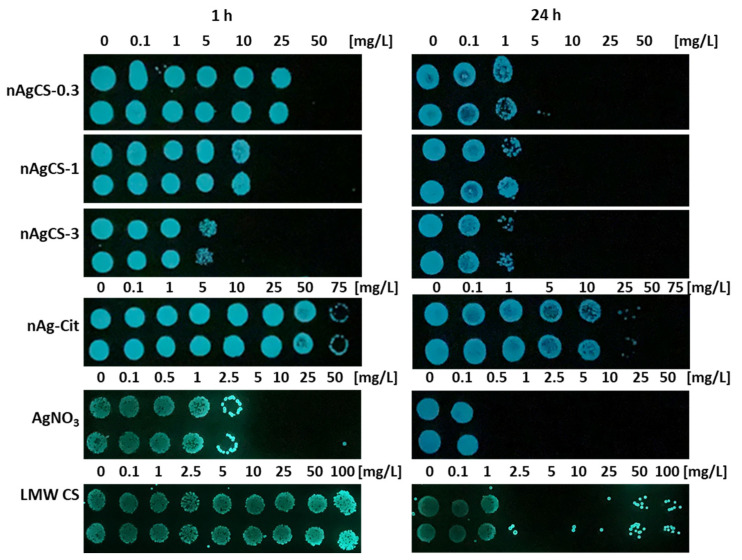
Colony forming ability (viability) of bacteria *Vibrio fischeri* on agar plates after 1 and 24 h exposure to the different concentrations of silver-chitosan nanocomposites with silver-chitosan weight ratios of 1:0.3 (nAgCS-0.3), 1:1 (nAgCS-1) and 1:3 (nAgCS-3), citrate-coated silver nanoparticles (nAg-Cit), AgNO_3_ and low molecular weight chitosan (LMW CS) in 2% NaCl solution. Please note that the EC_50_ values for chitosan are presented as mg compound/L and Ag-containing compounds as mg Ag/L. The photos were taken in the dark.

Interestingly, LMW CS inhibited *V. fischeri* bioluminescence almost immediately upon contact, showing that chitosan—a polycationic natural polymer—affects the integrity of bacterial membranes (Figure 2E). It is well known that the toxic effects of chitosan depend on pH (see below). The chitosan sample was tested at a slightly acidic pH (5.2–5.5) and exhibited rapid action. The 30-min EC_50_ value of chitosan, based on bioluminescence inhibition, was 5.6 mg/L. The inhibition of bioluminescence was a more sensitive parameter than bacterial growth inhibition by chitosan as the 1-h MBC value was notably higher, >100 mg/L (Figure 3). Thus, chitosan caused the rapid reduction/inhibition in bioluminescence but was less effective as a biocide according to the MBC values.

There is still a shortage of ecotoxicological information on chitosan. It is also important to note that chitosan dissolves in water under acidic conditions when the pH is below its pKa value of 6.5 [30]. Studies performed by Chou et al. [31] showed that LMW CS rapidly induced the death of zebrafish larvae and adults in water at 10–100 mg/L, even in normal river water and 24 h LC_50_ (survival rate) was 10–50 mg/L. In both cases, the toxicity was present at pH < 7 and disappeared when the samples were neutralized by phosphate buffer [31]. The *V. fischeri* test medium is 2% NaCl and thus contains no buffering components. Due to that, the pH values must be registered while observing toxic effects.

Comparison of the effects of nAgCSs and nAg–Cit on *V*. *fischeri* growth inhibition shows that nAgCSs and especially nAgCS-3 were remarkably more antibacterial than nAg-Cit (MBC values differed up to 10-fold) (Table 2 and Figure 3) and the toxicity of the nAgCSs depended on the silver-chitosan weight ratio. Indeed, the most toxic was the nAgCS-3, and the least toxic was the nAgCS-0.3; the respective 30-min EC_50_ values for the bioluminescence inhibition were 3.20 ± 2.0 and 25.6 ± 13.2 mg Ag/L (12.8 and 33.3 mg/L) (Table 2). The differences in these toxicity values were statistically significant (*p* < 0.05). Considering that the chitosan content in nAgCS-3 and nAgCS-0.3 was 9.60 and 8.45 mg/L, respectively, and the 30-min EC_50_ value for chitosan alone was 5.6 mg/L, the toxic effect can primarily be explainable by the chitosan content. Moreover, the shed Ag ions concentrations at the respective EC_50_ values were 0.12–0.82 mg Ag/L, which was ~13–90 times lower than the AgNO_3_ (Ag ions) 30-min EC_50_ value (Table 2). Interestingly, in the growth inhibition test at the respective 1-h MBC values for nAgCSs (Table 2), the chitosan content was 16.5–30.0 mg/L and solubilized silver ~0.36–1.6 mg Ag/L, which were lower than the corresponding 1-h MBC values for the given compounds (1-h MBC for AgNO_3_ and chitosan was 5 mg Ag/L and >100 mg/L, respectively), suggesting a synergistic biocidal effect of chitosan and silver in the studied nAgCSs.

Evaluating the toxic effects of silver compounds using the *V. fischeri* test is problematic as silver ions—the main drivers of the toxicity of silver compounds [2]—form a practically insoluble salt (AgCl) in 2% NaCl. Despite this, the 30-min EC_50_ value for AgNO_3_ was relatively low, 10.6 ± 4.9 mg Ag/L (Table 2), which is about 10-fold higher than the values reported by Kaiser and Devillers (1994) [25] for this assay, ranging from 0.44–1.48 mg Ag/L but comparable to EC_50_ value of 9.5 mg Ag/L reported by Hsieh et al. (2004) [32]. As mentioned above, the silver ions (AgNO_3_) did not exhibit a toxic response in the *V. fischeri* test within 15 s (Figure 2F), which is typical for toxic metals [29].

### 3.3. Ecotoxicity Evaluation of Studied Compounds to Aquatic Microcrustaceans

As shown in Table 2, all tested silver–chitosan nanocomposites (nAgCSs) and nAg-Cit were very toxic to aquatic microcrustaceans, showing adverse effects at the sub-mg per litre level. The toxicity of nAgCS-0.3, nAgCS-1 and nAgCS-3 to *T. platyurus* was statistically comparable when the concentrations were presented on a silver basis: the 24-h LC_50_ values ranged from 0.19–0.26 mg Ag/L. The pattern was similar for *D. magna*; the 48-h EC_50_ values for all nAgCSs were comparable but slightly lower, ~0.05–0.06 mg Ag/L. The toxicity of nAg-Cit was comparable for both crustacean species (about 0.11 mg Ag/L). These results indicate that the toxicity driver in the case of these nanocomposites towards aquatic crustaceans is silver. Indeed, when the toxicity values are presented on a compound basis, the silver-chitosan nanocomposites with a higher share of chitosan and, respectively, a lower share of silver were of lower toxicity to crustaceans (Table 2).

Toxicity evaluation of LMW chitosan to both tested crustacean species (*D. magna* and *T. platyurus)* showed no toxic effect at concentration ≤1 mg/L and L(E)C_50_ was about 12 mg/L. At a concentration of 10 mg/L, chitosan immobilized 35.3 ± 14.8% of *D. magna* and caused a 10–20% mortality of *T. platyurus*. Notably, the microcrustaceans exposed to 10 mg/L were entangled with chitosan, showing that at higher concentrations, chitosan may affect the viability of these test organisms mechanically. As the toxic effect of chitosan was observed at a much higher concentration than in the case of the Ag-chitosan nanocomposites, the toxicity of nAgCSs (Table 2) could be explained by released Ag-ions as it has been shown by various researchers for different types of AgNPs [2,33]. In the case of the nAgCS-3 (Ag-chitosan weight ratio 1:3), at the L(E)C_50_ values of 0.058 and 0.261 mg Ag/L (Table 2) for *D. magna* and *T. platyurus*, respectively, the chitosan content was 0.174 and 0.783 mg/L, being ~15–68 times lower than the chitosan L(E)C_50_ for the studied crustaceans. However, the toxicity of Ag-ions to both crustacean species used in this study was very high, with L(E)C_50_ values at 0.001–0.003 mg Ag/L (Table 2). Thus, the studied nAgCSs were about 60-fold less toxic than Ag-ions, and the shedding of Ag-ions from nAgCSs in the respective test media was about 1% (Table 1), explaining the primary mode of action of nAgCSs to the evaluated aquatic micro-crustaceans. The available scientific literature EC_50_ values for AgNO_3_ obtained from acute 48 h *D. magna* tests in different AFW vary from 0.4 to 6.0 µg Ag/L [34]. Such data variability may be explained by the chemical composition of the test medium/speciation [35]. In test media with a chemical composition similar to the one used in the current study, EC_50_ values 0.7–1.8 µg Ag/L [33,36,37] were very close to those reported in Table 2, confirming our test results.

**Figure 4:**
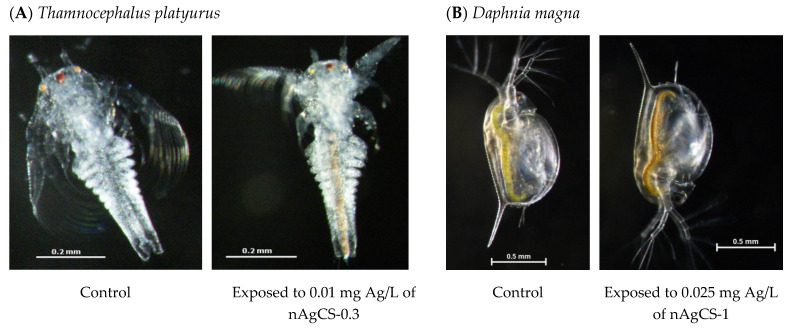
Accumulation of the silver-chitosan nanocomposites (nAgCSs) in the gut of alive particle-ingesting microcrustaceans: *Thamnocephalus platyurus* (**A**) and *Daphnia magna* (**B**). See also Table 2. A Nikon stereo microscope (SMZ1270) was used for imaging. Please note the different sizes of scale bars.

*D. magna* proved to be more sensitive to nAgCSs than *T. platyurus* (Table 2). This difference in sensitivity was not evident with nAg-Cit (Table 2), possibly explained by chitosan acting as a coating/stabilizer in nanocomposites, as well as the different sizes of the test organisms (Figure 4). The main exposure pathways of nanosilver for crustaceans are (i) contact with water/test media and (ii) ingestion. Both species have ingested nAgCSs during exposure (Figure 4). However, *D. magna* is larger than *T. platyurus* and can also ingest larger particles, which may remain unavailable to *T. platyurus*. Daphnids may ingest particles up to 70 μm in diameter [38]. Moreover, due to longer exposure time (48 h vs. 24 h), daphnids may ingest more nAgCSs agglomerates settled at the flask bottom than *T. platyurus*.

Last but not least, we performed the hazard ranking of studied chitosan-nanocomposites based on ecotoxicity classification as described in [2] and based on the toxicity results of the most sensitive toxicity test organism used. The hazard ranking categories: the median L(E)C_50_ value ≤ 1 mg/L = very toxic to aquatic organisms; >1–10 mg/L = toxic to aquatic organisms; >10–100 mg/L = harmful to aquatic organisms; >100 mg/L = not classified. Importantly, compound-based L(E)C_50_ values were used for hazard ranking.

According to the test results obtained from the multi-trophic test battery, where *D. magna* was the most sensitive test organism, the silver-chitosan nanocomposites were ranked from ‘extremely toxic’ [L(E)C_50_ ≤ 0.1 mg/L] to ‘very toxic’ [L(E)C_50_ > 0.1–1 mg/L]. The EC_50_ value of chitosan to *D. magna* was ~12 mg/L; thus, chitosan was ranked as ‘harmful’ [L(E)C_50_ > 10–100 mg/L]. Accordingly, silver-chitosan nanocomposites may pose a hazard to aquatic organisms and must be handled accordingly.

## 4. Conclusions

The data obtained in the current study show that the chitosan–silver nanocomposites could pose a risk to aquatic organisms and must be handled accordingly. The toxic effect of chitosan–silver nanocomposites on aquatic test organisms was explained mainly by the shedding of silver ions from these materials. Therefore, the environmental safety of chitosan-silver nanocomposites for freshwater ecosystems should be evaluated/predicted/regulated based on the shedding and toxicity of Ag-ions. However, it is unlikely that the amount of the nAgCSs targeted for use in biomedical applications will lead to significant environmental contamination as the amount of Ag used in the other sectors is more significant.

## Figures and Tables

**Table 1:** Physico-chemical properties of silver-chitosan nanocomposites (nAgCSs) and citrate-coated silver nanoparticles (nAg-Cit) in deionized (DI) water and toxicity testing media (bacteria: 2% NaCl and microcrustaceans: artificial fresh water, AFW) at 10 mg Ag/L. The solubility of silver compounds was determined at a concentration of 1 mg Ag/L after 1, 24 or 48 h incubation in toxicity testing media at ambient temperature (20 °C).

Tested Compounds	Ag:CS Weight Ratio	Primary Size ^a^ [nm]	ζ-Potential [mV]	Hydrodynamic Diameter D_hyd_^b^ [nm] and (PDI) ^c^			Solubility ^d^, µg Ag/L (%) ^e^		
				DI water	2% NaCl	AFW	1-h 2% NaCl	24-h AFW	48-h AFW
nAgCS-0.3	1:0.3	~50	+15.8	102 ± 8 (0.22)	113 ± 1 (0.29)	>1000	32.4 ± 6.6 (3.2%)	10.1 ± 1.0 (1.0%)	8.9 ± 1.5 (0.9%)
nAgCS-1	1:1	~50	+21.8	91.8 ± 9.6 (0.32)	92.2 ± 1.2 (0.27)	>1000	16.0 ± 2.9 (1.6%)	5.6 ± 0.3 (0.6%)	4.9 ± 0.5 (0.5%)
nAgCS-3	1:3	~50	+26.4	78.8 ± 7.3 (0.45)	110 ± 1 (0.29)	>1000	35.5 ± 13.7 (3.6%)	5.6 ± 0.4 (0.6%)	5.5 ± 0.3 (0.6%)
nAg-Cit	1:0	~50	−36.1	36.9 ± 3.9 (0.48)	248 ± 20 (0.30)	>1000	20.3 ± 3.7 (2.0%)	16.7 ± 0.5 (1.7%)	15.5 ± 0.3 (1.6%)

Notes: ^a^ data from scanning transmission electron microscopy (STEM) analysis [19]; ^b^ by the intensity of scattered light diameter; ^c^ PDI—polydispersity index, ^d^ atomic absorption spectroscopy (AAS) analysis; ^e^ solubility %.

**Table 2:** Toxicity of silver–chitosan nanocomposites (nAgCS), citrate-coated silver nanoparticles (nAg-Cit) and AgNO_3_ to crustaceans *Daphnia magna*, *Thamnocephalus platyurus* and bacteria *Vibrio fischeri*. L(E)_50_ or MBC (minimum bactericidal concentration) values are nominal and presented as mg Ag/L. Concentrations for low molecular weight chitosan (LMW CS) and silver compounds in the brackets are presented on the whole compound basis, mg/L. L(E)C_50_ values are presented as an average of a minimum of three replicates ± SD.

	Aquatic Microcrustaceans		Bacteria		
Test Species:	*Daphnia magna*	*Thamnocephalus platyurus*	*Vibrio fischeri*		
Tested Compounds	48-h EC_50_	24-h LC_50_	30-min EC_50_	1-h MBC	24-h MBC
nAgCS-0.3	0.050 ± 0.01 (0.065)	0.191 ± 0.02 (0.248)	25.6 ± 13.19 (33.3)	50 (65)	5 (6.5)
nAgCS-1	0.055 ± 0.02 (0.110)	0.224 ± 0.02 (0.448)	9.00 ± 4.62 (18.0)	25 (50)	5 (10)
nAgCS-3	0.058 ± 0.01 (0.232)	0.261 ± 0.02 (1.044)	3.20 ± 2.00 (12.8)	10 (40)	5 (20)
nAg-Cit	0.118 ± 0.02 (0.568)	0.112 ± 0.01 (0.539)	>75 (>360)	>75 (360)	50 (240)
AgNO_3_	0.001 ± 0.0005 (0.002)	0.003 ± 0.0007 (0.005)	10.6 ± 4.9 (16.8)	5 (7.94)	0.5 (0.79)
LMW CS	~12 *	~12 **	5.60 ± 3.52	>100	5

* 10 mg/L immobilized 35.3 ± 14.8% of *D. magna*; ** 10 mg/L caused 10–20% mortality of *T. platyurus*. The chitosan concentrations higher than 10 mg/L were not tested as the pH of exposure test solutions was below 7.0, which is not optimal for crustaceans.

## Data Availability

The original contributions presented in the study are included in the article, further inquiries can be directed to the corresponding author.
